# Molecular Influence of the ATM Protein in the Treatment of Human Cells with Different Radioprotective Drugs: Comparisons between Antioxidative and Pro-Episkevic Strategies

**DOI:** 10.3390/biom13030524

**Published:** 2023-03-13

**Authors:** Juliette Restier-Verlet, Michel Drouet, Pauline Pras, Mélanie L. Ferlazzo, Adeline Granzotto, Laurène Sonzogni, Joëlle Al-Choboq, Laura El Nachef, Sabine François, Michel Bourguignon, Nicolas Foray

**Affiliations:** 1 INSERM U1296 Unit “Radiation: Defense, Health, Environment”, Centre Léon-Bérard, 69008 Lyon, France; 2 Division Défense NRBC, Institut de Recherche Biomédicale des Armées, 91220 Brétigny-sur-Orge, France; 3 Department of Biophysics and Nuclear Medicine, Université Paris Saclay Versailles St Quentin en Yvelines, 78035 Versailles, France

**Keywords:** radioprotectors, N-acetylcysteine, amifostine, statin, bisphosphonates, ATM

## Abstract

The radiation protection strategy with chemical agents has long been based on an antioxidative approach consisting in reducing the number of radical oxygen and nitrogen species responsible for the formation of the radiation-induced (RI) DNA damage, notably the DNA double-strand breaks (DSB), whose subset participates in the RI lethal effect as unrepairable damage. Conversely, a DSB repair-stimulating strategy that may be called the “pro-episkevic” approach (from the ancient Greek *episkeve*, meaning repair) can be proposed. The pro-episkevic approach directly derives from a mechanistic model based on the RI nucleoshuttling of the ATM protein (RIANS) and contributes to increase the number of DSB managed by NHEJ, the most predominant DSB repair and signaling pathway in mammalians. Here, three radioresistant and three radiosensitive human fibroblast cell lines were pretreated with antioxidative agents (N-acetylcysteine or amifostine) or to two pro-episkevic agents (zoledronate or pravastatin or both (ZOPRA)) before X-ray irradiation. The fate of the RI DSB was analyzed by using γH2AX and pATM immunofluorescence. While amifostine pretreatment appeared to be the most efficient antioxidative process, ZOPRA shows the most powerful radiation protection, suggesting that the pro-episkevic strategy may be an alternative to the antioxidative one. Additional investigations are needed to develop some new drugs that may elicit both antioxidative and pro-episkevic properties and to quantify the radiation protection action of both types of drugs applied concomitantly.

## 1. Introduction

**Figure 1:**
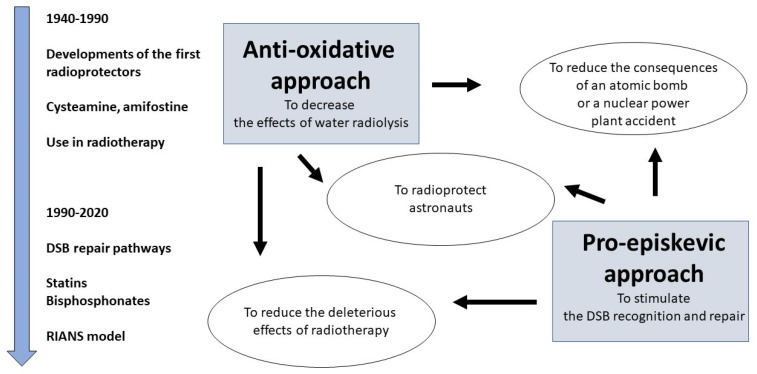
**Schematic view of the two different approaches of radioprotection, their major principles and their historical milestones**.

Exposure to ionizing radiation (IR) causes a large spectrum of DNA damage and cellular deaths and affects individuals by increasing risks of radiation-induced (RI) tissue/organ injuries (radiosensitivity), cancers (radiosusceptibility) and/or aging (radiodegeneration) [1]. Since the first studies initiated in 1942 by the scientists of the Manhattan Project at Walter Reed General Hospital (Washington, DC, USA) and by Antoine Lacassagne at Institut Curie (Paris, France), radiobiologists have endeavored to develop chemical countermeasures aiming to protect DNA, cells, tissues and individuals from the clinical consequences of exposure to IR [2,3,4,5,6,7]. In the field of radiation protection, specific terms are currently used to describe the different radioprotective agents, whether they are administered before (prophylactic drugs), during or after (mitigation drugs) irradiation [2]. The radioprotective agents should be efficient in different scenarios, such as after accidental, military, occupational and clinical exposures to IR (Figure 1):

- High-intensity warfare has recently emerged as a reality in Europe: Old cold war challenges are currently largely under review, among which the place of radioprotector countermeasures should usefully be revisited. Military operations under radiological/nuclear threat (even limited to tactical level) could result in numerous cases of acute radiation syndrome. Targeting fallout may be more realistic and could offer opportunities for innovative radioprotectors use. As an example, nuclear power plant security during military operations has been a concern for some years in NATO strategy, and recent operations in Ukraine (Tchernobyl area and Zaporizhia power plant) clearly illustrate some of these various scenarios. In such context, maintaining the freedom of action of forces may result in deliberate or accidental limited irradiation of soldiers up to 750 mGy [8].
- Space radiation represents the most hazardous factor of space exploration [9]. For the astronauts in their spacecraft, exposure to space radiation can be summarized as a continuous low dose rate of X- and gamma-rays (the “bath of radiation”) and a flux of low-energy particles emitted from the metallic shielding [9]. Hence, specific radiation protection countermeasures are needed to protect astronauts.
- Radioprotective agents are useful in the medical context, notably in anticancer radiotherapy (RT) to specifically protect healthy tissues surrounding the tumor [2,3,10,11]. There has been a plethora of RT trials involving radioprotectors, generally sulfhydryl compounds and other antioxidants. Analogs of cysteine, notably cysteamine, and glutathione were applied to irradiated animals and then cancer patients; the development of new thiol-containing agents was a major axis of research in this field [12,13,14,15,16]. More than 4000 candidate compounds were tested in the Walter Reed Army Research Center (USA) [7]. Among them, WR2721 (ethyol and amifostine) appeared to be the most efficient radioprotective drug in RT. Amifostine was notably shown to reduce IR-induced esophagitis, mucositis, lung inflammation and cisplatin-related nephrotoxicity. Despite some side effects, the application of amifostine during RT generally permits to achieve high rates of complete response [17,18,19,20,21,22,23]. Since 1999, the use of amifostine in RT is permitted by the US Federal Drug Agency agreement to reduce xerostomia in patients undergoing postoperative RT for head and neck cancer [21].

In addition to amifostine, a number of antioxidants such as glutathione, vitamin E, selenium or N-acetylcysteine (NAC) have been used concomitantly with chemo- and radiotherapy [2,3,24]. Among the antioxidants, NAC is one of the most extensively used and provides the advantage of a low cost and the absence of toxicity. The major action of NAC is the increase in glutathione levels, which facilitates the prevention against the pathologies linked to free radicals and reactive species [25]. Mainly supported by experiments on exposed rats, first RT trials with NAC have revealed a significant heart and lung protection [26,27]. However, whatever the antioxidants tested, the experimental protocols are so various that a very small minority of trials involved the same drug, which does not permit to conclude a real benefit of any antioxidative drug during anticancer RT, and reciprocally, it does not facilitate reliable intercomparisons between the different antioxidants [2,3] (Figure 1).

To date, the great majority of the studies about radioprotectors follow the same *antioxidative* approach, based on the reduction in the ROS levels that produce RI DNA damage. Consistently, this approach results in decreasing the number of DNA breaks, notably that of DNA double-strand breaks (DSB), the key damage of the cell killing [28,29]. An alternative approach would consist rather in *stimulating DSB repair* than *reducing the number of the RI DSB*. Such a strategy has been named the *pro-episkevic* approach (from the ancient Greek επισκευε (episkeve), meaning “repair”). This term was first mentioned in the PhD thesis dissertation of M.L.F presented in 2017 at the University of Lyon, France [30]. The pro-episkevic approach has been mainly documented in the frame of the RI nucleoshuttling of the ATM protein (RIANS) model [31,32,33,34]. The ATM protein is a major actor in the individual response to IR and DNA-breaking agents. The RIANS model is based on the following molecular steps of the stress response of quiescent cells: (1) RI oxidative stress results in both induction of DSB in nucleus and monomerization of the cytoplasmic ATM dimers; (2) the resulting ATM monomers diffuse from cytoplasm to nucleus; (3) the ATM monomers phosphorylate H2AX histones, which produces nuclear γH2AX foci at the DSB sites (easily visible and quantifiable by immunofluorescence) and contribute to the recognition of DSB by the nonhomologous end-joining (NHEJ) pathway, predominant in human quiescent cells [35,36,37]; (4) a complete DSB repair produces the trans-autophosphorylation of ATM proteins (pATM) that forms ATM dimers in nucleus, reflected by pATM foci at the DSB sites, again easily visible and quantifiable by immunofluorescence [34,36,38,39]. A delayed RIANS may be caused by an overexpression of some ATM phosphorylation substrate proteins (called X-proteins) in cytoplasm. The cytoplasmic X-proteins sequestrate the ATM monomers. As a consequence, either the *unrecognized* DSB remain unrepaired and participate in cell death, or else, they are *misrepaired* by error-prone recombination-like pathways and participate in cell transformation and cancer [34,36,38,39]. A combination of statins and bisphosphonates was shown to accelerate the RIANS by increasing the permeability of the nuclear membranes to the ATM monomers and to provide various levels of radioprotection according the genetic syndromes tested [31,32,33,34,40,41] (Figure 1).

Recently, from 200 human fibroblast cell lines showing a large spectrum of radiosensitivity, we provided clues that some quantitative correlations exist between the cellular radiosensitivity of quiescent human cells and γH2AX and pATM biomarkers [42]. Other biomarkers such as DNA-PK foci, Ku expression, or cell-free plasmid end-joining assays were not chosen because of potential technical artifacts, low dose dependence and difficulties to extrapolate data to in vivo experiments, respectively [1,42,43]. Hence, here, by using the number of γH2AX and pATM foci as endpoints, the antioxidative approach (with NAC and amifostine, the most documented antioxidative drugs) and the pro-episkevic approach (with statins and bisphosphonates, the most documented pro-episkevic drugs) were compared with three radioresistant and three radiosensitive human quiescent fibroblasts, pretreated with drug concentrations already tested in the literature and exposed to IR. Our major goal is to better understand the molecular features of the radioprotection process by quantifying the reduction of the molecular effect induced by a reference IR dose (here, 2 Gy X-rays, mimicking a standard RT session) after a 24 h drug pretreatment.

## 2. Materials and Methods

### 2.1. Cell Lines

All the skin fibroblasts tested were untransformed to avoid any bias linked to genomic stability in the plateau phase of growth (95–99% in G0/G1) to overcome any cell cycle and homologous recombination effects. Skin fibroblasts were routinely cultured at 37 °C in 5% CO_2_ humid conditions as monolayers with Dulbecco’s modified Eagle’s minimum medium (DMEM) (Gibco-Invitrogen-France, Cergy-Pontoise, France), supplemented with 20% fetal calf serum, penicillin and streptomycin. The origin and the radiobiological features of the RIANS-normal radioresistant AG1521, 1BR3 and 149BR controls are published elsewhere [39,44,45]. The radiosensitive RIANS-delayed 09CLB, 11CLB and 15CLB fibroblasts were provided from skin biopsy from donors who show adverse tissue reaction after anticancer RT. These cell lines belong to the “COPERNIC” collection managed by our lab and approved by the regional Ethical Committee. The COPERNIC cell lines were declared under the numbers DC2008-585, DC2011-1437 and DC2021-3957 to the Ministry of Research. The radiobiological database was protected under the reference as IDDN.FR.001.510017.000.D.P.2014.000.10300 [39]. The surviving 2 Gy (SF2) of the cell lines used in this study is assessed and published elsewhere [42]. Briefly, while the SF2 of the radioresistant AG1521, 1BR3 and 149BR controls ranged between 60 and 65%, the SF2 of 09CLB, 11CLB and 15CLB fibroblast cell lines were found to be 35 ± 5, 42 ± 8 and 37 ± 4%, respectively. It is noteworthy that 09CLB, 11CLB and 15CLB donors suffered from severity grades 4, 3 and 4 tissue post RT reaction [39,42].

### 2.2. Radioprotective Drugs

With regard to the antioxidative drugs, N-acetyl-L-cysteine (NAC) and amifostine (2-(3-Aminopropyl)aminoethyl phosphorothioate, WR2721, Ethyol) were purchased from Sigma-Aldrich (Saint-Quentin-Fallavier, France; #9165 and #A5922, respectively). Cells were incubated with these two last drugs separately for 24 h at 37 °C at 10–300 mM NAC or at 24–96 mM for amifostine. The choice of these drug concentrations was motivated by preliminary experiments based on the assessment of γH2AX foci when drug was applied alone to radioresistant fibroblasts. All the conditions leading to the production of more than 2 γH2AX foci per cell were excluded. With regard to the pro-episkevic drugs, cells were incubated with either 1 µM pravastatin (#P4498, Sigma-Aldrich) for 24 h at 37 °C alone or 1 µM zoledronate (#SML0223, Sigma-Aldrich) for 12 h at 37 °C alone (to permit intercomparisons) or together (ZOPRA treatment) [46]. The choice of these drug concentrations was notably motivated by our previous data published in [46].

### 2.3. X-ray Irradiations

Irradiations were performed with a 6 MeV X-ray medical irradiator (SL 15 Philips) (dose rate: 6 Gy.min^−1^) at the anticancer Center Léon-Bérard (Lyon, France) [39,45]. In all the experiments, a dose of 2 Gy was applied. It is noteworthy that 2 Gy X-rays represent a reference dose equivalent to a session of a standard anticancer RT.

### 2.4. Immunofluorescence

The immunofluorescence protocol was described elsewhere [47,48]. Briefly, cells were fixed in paraformaldehyde for 10 min at room temperature and were permeabilized in 0.5% Triton X-100 solution for 5 min at 4 °C. Primary and secondary antibody incubations were performed for 40 and 20 min at 37 °C, respectively. Anti-*γH2AX^ser139^* antibody (#05636; Upstate Biotechnology-Euromedex, Mundolsheim, France) was used at 1:800. The monoclonal anti-mouse anti-*pATM^ser1981^* (#ab2888) from Abcam (Cambridge, UK) was used at 1:100. Incubations with anti-mouse fluorescein (FITC) and rhodamine (TRITC) secondary antibodies were performed at 1:100 at 37 °C for 20 min. Slides were mounted in 4′,6′ Diamidino-2-Phenyl-indole (DAPI)-stained Vectashield (Abcys, Paris, France), and foci were examined with Olympus fluorescence microscope. The foci scoring procedure applied here received the certification agreement of CE mark and ISO-13485 quality management system norms. Our foci scoring procedure also developed some features that are protected in the frame of the Soleau Envelop and patents (FR3017625 A1, FR3045071 A1, EP3108252 A1) [48]. More than 50 nuclei were analyzed per experiment and at least 3 independent replicates were performed for each condition [39]. It is noteworthy that *γH2AX* foci reveal only the presence of DSB recognized by the NHEJ pathway: vis-à-vis the DSB induced “physically” by IR, the proportion of DSB recognized by NHEJ is maximal in radioresistant cells, while the number of *γH2AX* foci may be lower than the total number of DSB induced in radiosensitive cells, notably in those eliciting a delayed RIANS [38,39,42].

### 2.5. Statistical Analysis

Statistical significance between data points was verified with the one-way ANOVA test. Statistical analysis was performed by using Kaleidagraph v4 (Synergy Software, Reading, PA, USA).

## 3. Results

### 3.1. Radioprotective Effect of the Antioxidative N-Acetylcysteine (NAC)

**Figure 2:**
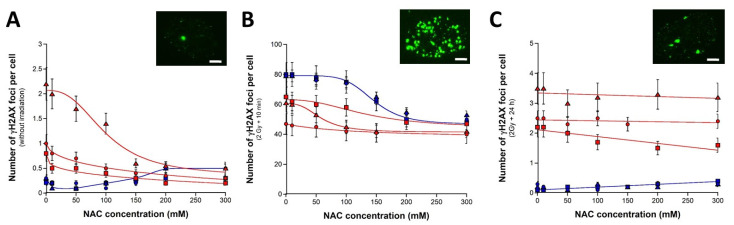
**Radioprotective effect of a pretreatment with NAC.** Number of γH2AX foci per cell in the human untransformed radioresistant AG1521 (blue squares), 1BR3 (blue triangles) and 149BR (blue circles) fibroblasts in the radiosensitive 09CLB (red triangles), 11CLB (red squares) and 15CLB (red circles) fibroblasts after pretreatment with NAC (**A**), followed by 2 Gy X-rays and 10 min (**B**) or 24 h (**C**) incubation. Each point represents the mean ± standard error (SEM) of at least three replicates. Sigmoidal or linear functions were used for data fitting. Inserts show representative examples of 300 mM pretreated 09CLB nuclei with γH2AX foci assessed at the indicated conditions. White bar represents 5 µm.

Before irradiation, while NAC did not significantly impact the number of spontaneous γH2AX foci in radioresistant controls below 150 mM, the NAC pretreatment decreased the number of γH2AX foci in all the radiosensitive fibroblasts tested (*p* < 0.001, *p* < 0.01 and *p* < 0.05 for the 09CLB, 11CLB and 15 CLB fibroblasts, respectively) (Figure 2A). Interestingly, the effect of NAC on radiosensitive cells fitted well with a sigmoidal curve (all the correlation coefficients’ r were found higher than 0.8) that converged to an average minimal value of 0.3 ± 0.1 γH2AX foci per cell (0.1 ± 0.1 γH2AX foci per cell for the radioresistant controls, *p* < 0.05). These findings suggest that millimolar concentrations of NAC may decrease the number of spontaneous DSB (Figure 2A).

When the number of γH2AX foci was assessed 10 min post irradiation, the untreated radioresistant controls showed an average of 79.5 ± 1.5 γH2AX foci per cell, in agreement with the literature data and with a DSB induction rate of 37 ± 4 γH2AX foci per Gy per cell (*p* > 0.8) [45] (Figure 2B). After NAC pretreatment, the number of γH2AX foci assessed 10 min post irradiation was shown to decrease significantly by obeying a sigmoidal function of the NAC concentration in all the radioresistant controls. In these cases, all the correlation coefficients’ r were found higher than 0.9, and the lowest average value corresponded to 50.3 ± 1.5 γH2AX foci. The radiosensitive fibroblasts showed a significantly lower number of γH2AX foci without NAC pretreatment than radioresistant controls (*p* < 0.01), according to previous data [39]. The NAC pretreatment led to decrease the number of γH2AX foci assessed 10 min post irradiation, suggesting that millimolar concentration of NAC may significantly decrease the number of DSB induced by IR *and* recognized by NHEJ. However, the lowest average number of γH2AX foci reached with radiosensitive cells was not found significantly different from that reached by radioresistant controls (*p* > 0.1) (Figure 2B).

Finally, the number of γH2AX foci assessed 24 h post irradiation was examined. As previously reported, the numbers of γH2AX foci assessed 24 h post irradiation were found not significantly different from zero in the untreated radioresistant controls (*p* > 0.5) (Figure 2C). The NAC pretreatment did not significantly change these values (*p* > 0.3). Interestingly, this last conclusion was also reached with the 09CLB and 15CLB radiosensitive fibroblasts (*p* > 0.3), even if the γH2AX foci values were significantly higher than those found with the radioresistant controls (*p* < 0.01). In the radiosensitive 11CLB fibroblasts, the NAC pretreatment was found to decrease significantly the number of γH2AX foci assessed 24 h post irradiation (*p* < 0.01) (Figure 2C). Altogether, these data suggest that the NAC pretreatment may produce a significant radioprotective effect on spontaneous DSB, as well as on a subset of recognized DSB (Figure 2).

### 3.2. Radioprotective Effect of the Antioxidative Amifostine (Ethyol)

**Figure 3:**
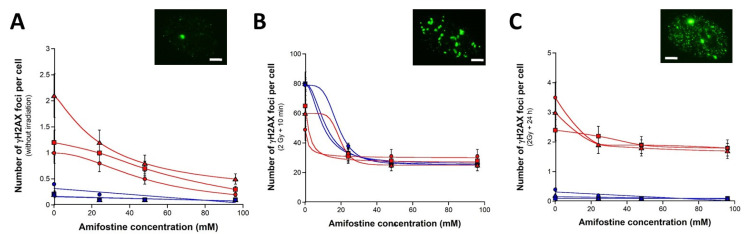
**Radioprotective effect of a pretreatment with amifostine.** Number of γH2AX foci per cell in the human untransformed radioresistant AG1521 (blue squares), 1BR3 (blue triangles) and 149BR (blue circles) fibroblasts and in the radiosensitive 09CLB (red triangles), 11CLB (red squares) and 15CLB (red circles) fibroblasts after pretreatment with amifostine (**A**), followed by 2 Gy X-rays and 10 min (**B**) or 24 h (**C**) incubation. Each point represents the mean ± standard error (SEM) of at least three replicates. Exponential, sigmoidal or linear functions were used for data fitting. Inserts show representative examples of 96 mM pretreated 09CLB nuclei with γH2AX foci assessed at the indicated conditions. White bar represents 5 µm.

Fibroblasts were pretreated to 24–96 mM amifostine for 24 h and irradiated thereafter. Despite a systematic decrease with concentration, the amifostine pretreatment did not significantly impact the number of spontaneous γH2AX foci in the radioresistant controls (*p* > 0.2) (Figure 3A). Conversely, in the radiosensitive fibroblasts, the amifostine pretreatment resulted in a significant decrease in the number of spontaneous γH2AX foci that obeyed a decreasing exponential function of the amifostine concentration for the 09CLB cells (r = 0.9) and a sigmoidal function for the two other radiosensitive cell lines tested (r = 0.8) for both. These findings suggest that millimolar concentrations of amifostine may decrease the number of spontaneous DSB recognized by NHEJ in radiosensitive cells (Figure 3A).

The number of γH2AX foci assessed 10 min post irradiation was shown to decrease significantly by obeying a sigmoidal function of the amifostine concentration in all the fibroblasts tested (all the correlation coefficients’ r were found higher than 0.8) (Figure 3B). The lowest γH2AX foci reached 96 mM amifostine and corresponded to an average of 26 ± 0.5 and 28 ± 2 γH2AX foci per cell for the radioresistant and radiosensitive cells, respectively (*p* > 0.4). Interestingly, this value was found significantly lower than that obtained with the NAC pretreatment (*p* < 0.001). These findings support that amifostine results in significantly decreasing the number of DSB induced by IR and recognized by NHEJ (Figure 3B).

Lastly, the amifostine pretreatment did not significantly change the number of γH2AX foci assessed 24 h post irradiation in the radioresistant controls (*p* > 0.5) (Figure 3C). Conversely, the amifostine pretreatment led to a significant decrease in the number of γH2AX foci in the 09CLB and 15CLB radiosensitive cell lines (*p* < 0.04) from 26 mM and in the 11CLB cell lines (*p* < 0.05) at 96 mM amifostine (Figure 3C). In these conditions, the action of amifostine was found more efficient for the three radiosensitive cell lines tested than with NAC (*p* < 0.03). Altogether, these data suggest that amifostine pretreatment provides a radioprotective effect on spontaneous DSB, a reduction in the number of DSB recognized by NHEJ in a larger extent than with NAC pretreatment and a significant reduction in the remaining unrepaired DSB (Figure 3).

### 3.3. Radioprotective Effect of the Pro-Episkevic Drugs Zoledronate and Pravastatin

The findings described above showed that the pretreatment with NAC or amifostine antioxidative agents consisted in decreasing the number of γH2AX foci, likely because they contribute to reduce the ROS and therefore the number of DSB induced by IR in general. Such reduction is likely to be performed chemically, i.e., independently of any DSB repair and signaling pathway recognizing the DSB induced by IR. Conversely, pro-episkevic drugs such as zoledronate and pravastatin accelerate the RIANS and therefore increase the number of DSB recognized by NHEJ [34]. Hence, as far as the γH2AX foci reflect the DSB managed by NHEJ, we can expect an increase in the number of DSB recognized by NHEJ (i.e., early γH2AX foci), while the number of DSB “physically” induced by IR should remain constant.

**Figure 4:**
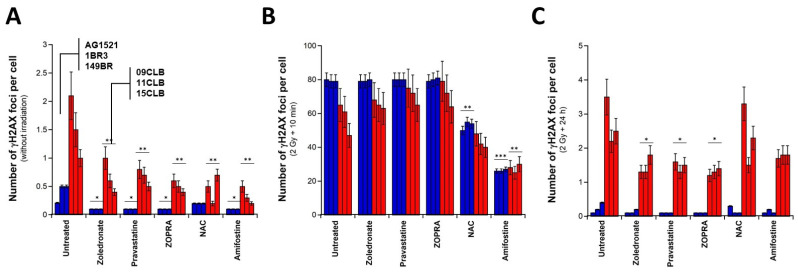
**Radioprotective effect of a pretreatment with zoledronate, pravastatine and ZOPRA.** Number of γH2AX foci per cell assessed in the indicated pretreatment and after the indicated pretreatment (**A**), followed by 2 Gy X-rays and 10 min (**B**) or 24 h (**C**) incubation. Each bar represents the mean ± standard error (SEM) of at least three replicates. The NAC and amifostine data are those already shown in Figure 3 and Figure 4 with pretreatment of 300 mM NAC and 96 mM amifostine. one, two and three asterisks indicate *p* < 0.05, *p* < 0.01 and *p* < 0.001, respectively, by comparison with untreated controls.

The fibroblasts were pretreated to zoledronate and pravastatin separately, as well as with the combination of both drugs (ZOPRA). In the radioresistant controls pretreated with zoledronate and pravastatin separately or concomitantly, the number of γH2AX foci assessed before irradiation was found to be significantly lower than that observed in the non-pre-treated cells (*p* < 0.03) (Figure 4A). The same value was reached whether cells were pretreated with zoledronate or pravastatin, taken separately or with ZOPRA (*p* > 0.8). The extent of such a decrease is the same as that found with the pretreatment with amifostine (*p* > 0.1). In radiosensitive cells, the picture changed, since more DSB recognized by NHEJ are expected: 0.5 ± 0.1 γH2AX foci vs. 0.3 ± 0.1 γH2AX foci per cell were found on average with the pro-episkevic drugs and the antioxidative drugs, respectively (*p* < 0.01). Altogether, these data suggest that zoledronate, pravastatin and ZOPRA may significantly reduce the number of spontaneous DSB but not to the same extent as antioxidative agents such as NAC or amifostine (Figure 4A).

In the radioresistant controls, the pretreatment of cells with zoledronate and pravastatin, separately and ZOPRA, has no significant effect on the number of γH2AX foci assessed 10 min post irradiation (*p* > 0.8), in agreement with the hypothesis that all the RI DSB are recognized by NHEJ in radioresistant cells (Figure 4B). Conversely, in the radiosensitive fibroblasts, the number of γH2AX foci assessed 10 min post irradiation increased with the pro-episkevic drugs pretreatment (by comparison with the untreated conditions), but this tendency was not significant (*p* < 0.08). The ZOPRA combination provided the largest increase by reaching the values of the radioresistant controls for the 09CLB and 11CLB cell lines. Such a tendency was drastically different from the reduction in the number of γH2AX foci assessed 10 min post irradiation observed with antioxidative agents, (*p* > 0.5 with NAC and *p* > 0.8 with amifostine). These findings suggested that the pro-episkevic drugs increase the number of DSB recognized by NHEJ among the DSB physically induced by IR, while the antioxidative drugs decrease the number of all the DSB physically induced by IR (Figure 4B).

By comparing it with data from untreated cells, the number of γH2AX foci assessed 24 h post irradiation in radioresistant controls did not change when pretreated with the pro-episkevic drugs, whether the drugs were applied separately or concomitantly (*p* > 0.8). By contrast, in radiosensitive cells, the number of γH2AX foci assessed 24 h post irradiation decreased significantly with pro-episkevic drugs (*p* < 0.03 with NAC and *p* < 0.04 with amifostine) and reached its lowest values with ZOPRA pretreatment (Figure 4C).

**Figure 5:**
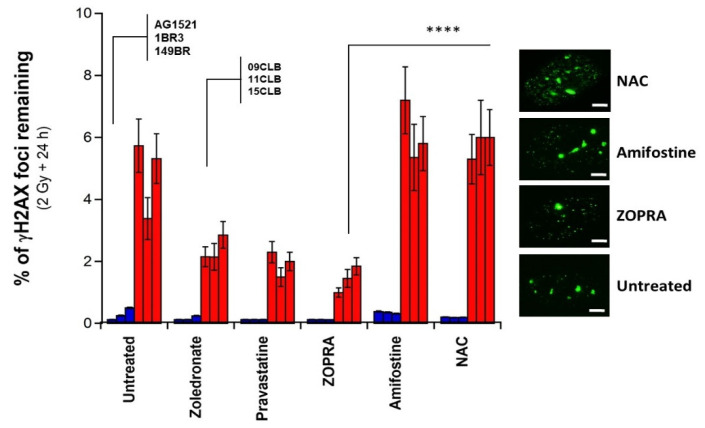
**Relative radioprotective effect of a pretreatment with zoledronate, pravastatine, ZOPRA, NAC and amifostine.** The data shown in Figure 3C are expressed as percentage of γH2AX foci remaining, calculated by dividing the number of γH2AX foci assessed 24 h post irradiation (Figure 4C) by the number of γH2AX foci assessed 10 min post irradiation (Figure 4B). Inserts show representative examples of untreated and ZOPRA, 300 mM amifostine and 96 mM NAC pretreated 09CLB nuclei with γH2AX foci assessed at the indicated conditions. White bar represents 5 µm. Four asterisks indicate *p* < 0.0001, respectively, between ZOPRA data and NAC and amifostine data.

To better illustrate such differences, the percentage of γH2AX foci remaining at 24 h post irradiation was calculated by dividing the number of γH2AX foci assessed 24 h post irradiation by the number of γH2AX foci assessed 10 min post irradiation. The resulting percentage was found to be the lowest with the ZOPRA pretreatment and the highest with the antioxidative drugs. Considering such percentage resulted in increasing the differences between ZOPRA on one hand and NAC and amifostine on the other hand (*p* < 0.001). These findings suggested that, even if NAC and amifostine may reduce the DSB physically induced by IR, they do not reduce the severity of the DSB remaining (Figure 5). Let us recall again that γH2AX foci represent the DSB recognized by the ATM-dependent NHEJ pathway [34], that the pro-episkevic drugs act on the NHEJ pathway specifically and that the antioxidative agents act on all the DSB, whatever the pathway involved.

### 3.4. Influence of the ATM Protein in the Antioxidative and Pro-Episkevic Approaches

**Figure 6:**
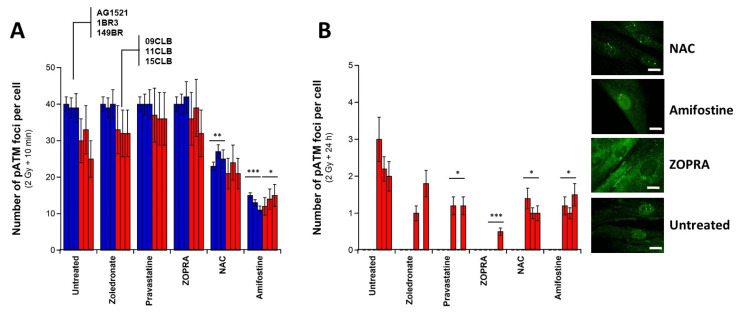
**Radioprotective effect of a pretreatment with zoledronate, pravastatine and ZOPRA assessed with pATM foci.** Number of pATM foci per cell assessed in the indicated pretreatment and after the indicated pretreatment, followed by 2 Gy X-rays and 10 min (**A**) or 24 h (**B**) incubation. Each point represents the mean ± standard error (SEM) of at least three replicates. Inserts show representative examples of untreated and ZOPRA, 300 mM amifostine and 96 mM NAC pretreated 09CLB nuclei with pATM foci assessed at 24 h post irradiation at the indicated conditions. White bar represents 10 µm. one, two and three asterisks indicate *p* < 0.05, *p* < 0.01 and *p* < 0.001, respectively, by comparison with the untreated controls.

In the frame of the RIANS model, the cytoplasmic ATM monomers diffuse to the nucleus and contribute to the recognition of DSB via NHEJ by phosphorylating H2AX molecules at the DSB sites [34]. Once the recognition and DSB repair steps are completed, the ATM monomers reassociate via a trans-autophosphorylation responsible of the formation of pATM foci [38,39]. We therefore examined the different action of the antioxidative NAC and amifostine and the pro-episkevic ZOPRA pretreatments by using pATM immunofluorescence. No spontaneous pATM focus was observed, whatever the conditions (data not shown). By contrast, the number of pATM foci assessed 10 min post irradiation reached similar conclusions as that with γH2AX foci, with the lowest values obtained with the pretreatment with the antioxidative drugs and the highest values with the pretreatment with ZOPRA (Figure 6A). The 24 h data reached similar conclusions as those reached with γH2AX data, with a nearly complete absence of pATM foci in ZOPRA pretreated cells (*p* < 0.001 by comparison with untreated conditions) and lower reduction with NAC and amifostine pretreatment (*p* < 0.04 by comparison with untreated conditions) (Figure 6B).

### 3.5. Review of the Radioprotective Action of the Pro-Episkevic ZOPRA Combinations

**Figure 7:**
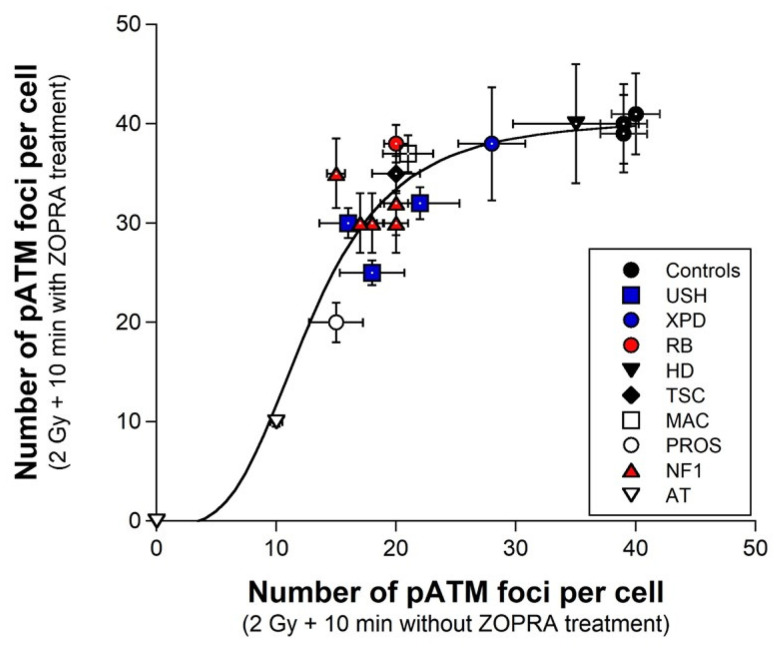
**Radioprotective effect of ZOPRA in fibroblasts from 10 genetic syndromes with pATM foci.** The number of pATM foci assessed at 10 min post irradiation (X-rays) with and without ZOPRA pretreatment were plotted together. Fibroblasts were derived from the indicated syndromes: Usher’s syndrome (USH), PROS syndrome (PROS), McCune–Albright syndrome (MAC), retinoblastoma (RB), Tuberous Sclerosis Complex syndrome (TSC), Huntington Disease (HD), Xeroderma Pigmentosum D (XPD), Neurofibromatosis 1 (NF1), LIG4 syndrome (LIG4) and ataxia telangiectasia (AT). Note the AT data are obtained from historical data of the N.F. lab; all these data were published elsewhere [31,32,33,40,41,48,49,50]. Each point represents the mean ± standard error (SEM) of at least three replicates. Data were fitted to the following sigmoidal function: y = 40.53 + (40.10)/(1 + (x/12.797)^3.51^); r = 0.944.

Since 2008, our research group has endeavored to document the major radiobiological features of genetic syndromes associated with radiosensitivity [43]. For the first time, by focusing on the pATM data only, we gathered the data involving ZOPRA pretreatment in 10 diseases, namely Usher’s syndrome (USH) [41], PROS syndrome (PROS) [49], McCune-Albright syndrome (MAC) [33], retinoblastoma (RB) [50], Tuberous Sclerosis Complex syndrome (TSC) [32], Huntington Disease (HD) [40], Xeroderma Pigmentosum D (XPD) [48], Neurofibromatosis 1 (NF1) [31], LIG4 syndrome (LIG4) [51] and ataxia telangiectasia (AT) [52]. When the numbers of pATM foci assessed at 10 min post irradiation with and without ZOPRA pretreatment were plotted together, a sigmoidal function appeared, indicating that there is no significant ZOPRA protective effect for cells eliciting fewer than 10 pATM foci per cell, such as the two *ATM*-mutated hyper-radiosensitive cell lines (Figure 7). Furthermore, for fibroblasts showing more than 10 but fewer than 25 pATM foci at 10 min post irradiation, the ZOPRA protective effect was found proportional to the number of pATM foci (Figure 7). From 30 pATM up to the maximal 40 pATM foci values, the ZOPRA protective effect reached a plateau (*p* > 0.8) (Figure 7). The sigmoidal function indicated that the maximal ZOPRA effect can add 15 additional recognized DSB to a number of 20 DSB already managed by NHEJ, which corresponds to a relative increase of 75%. Altogether, these findings suggest that ZOPRA cannot correct, on one hand, the hyper-radiosensitive cells with no or nearly absent nuclear ATM kinase activity, and on the other hand, the hyper-radiosensitive *LIG4*-mutated cells and radioresistant controls that all show a normal ATM kinase activity. Such data therefore support that the ZOPRA pretreatment is particularly adapted to correct moderate but significant radiosensitivity. Additional investigations are needed to identify more efficient pro-episkevic drugs and to ask whatever the combination of antioxidative and pro-episkevic agents may lead to an enhanced protection against IR.

## 4. Discussion

### 4.1. Documented Evidence That NAC and Amifostine May Act as Radioprotectors

An exposure to IR triggers the formation of a large spectrum of reactive oxygen and nitrogen species [53]. The natural antioxidative agents produced in cells are mostly related to glutathione (GSH) [54]. However, at high IR dose, the levels of endogenous RI-induced GSH molecules may be not sufficient to provide a significant radioprotective effect: exogenous thiols are required. A plethora of antioxidative thiol compounds have been developed to serve as countermeasures of IR-induced toxicity. Among them, NAC and amifostine appear as the most efficient radioprotectors [2]. NAC has been recommended as mucolytic and against acetaminophen overdose [55,56]. The great majority of radiobiological studies with NAC have been conducted with rats [2,3,24]. However, its radioprotective effect has also been documented in RT trials, notably in non-small-cell lung cancers [27]. For example, the pretreatment with NAC was shown to improve anastomoses and wound healing, to decrease post-irradiation toxicity and show anti-inflammatory effects in the liver, gastrointestinal tract, cochlea, bones and skin [55,56]. NAC has the double advantage of being cheap and to present few side effects. Historically, NAC is one of the simplest molecules proposed in the group of cysteine-containing agents. However, in the present study, the radioprotective effect of NAC was found to be lower than amifostine, the most extensively used radioprotector [2]. The actions of these two drugs have often been compared [24,57,58]. NAC may have two concomitant actions: (1) A reduction effect through which the free SH-groups of NAC break the disulfur R-S-S-R’ bridges of different proteins. As indicated above, such hydrolysis is exploited in gastric mucus to provide mucolytic properties [59]. (2) A cysteine production effect through which the deacetylation of NAC produces cysteine required for GSH production [60]. Conversely, amifostine is transformed to an active thiol when dephosphorylated by alkaline phosphatases, which is very abundant in the cellular membranes, to provide an SH group. Its two amine functions and SH permit a high power of chelation of ions, including Ca^2+^ ions, particularly produced after exposure to IR [61]. These various action modes of NAC and amifostine may explain some discrepancies in their radioprotective effect, likely due to tissue specificities and differences in experimental protocols. For example, by using clonogenic cell surviving fraction and yields of γH2AX foci as endpoints, Kataoka et al. (2007) systematically showed that amifostine was a better radioprotector than NAC in a human endothelial cell line [58]. Conversely, NAC was reported to be a better cytoprotective drug than amifostine in methotrexate-treated hepatic rat tissues [62]. Seker et al. did not find differences between the two drugs in RT-induced uterine tissue injuries in rats [63]. Despite such discrepancies, amifostine remains the only radioprotective agent approved in RT by FDA [2]. In this study, the pretreatment with 96 mM amifostine was found to be more efficient to protect cells against IR injuries than the most efficient concentration of NAC. It is also important to mention that amifostine (WR2721) is the thiophosphate prodrug of an active thiol form (WR1065) that is usually applied in culture medium at lower concentrations and that shows a radioprotective effect earlier than amifostine [2,64]. However, we deliberately chose to apply the drug recommended by the FDA as a 24 h pretreatment, as with the other drugs tested here, to facilitate intercomparisons.

Finally, with regard to the concentrations used, while it must be stressed that the drug concentrations used in ZOPRA treatment correspond to the posology applied in standard antiosteoporosis and anticholesterolemiant treatments [46], the question of translation to clinical use is more complex with regard to antioxidative drugs, even if the concentrations applied here have been extensively used in the literature and even if they currently present few side effects [2].

### 4.2. Documented Evidence That Statins and Bisphosphonates May Act as Radioprotectors

Pravastatin is currently used as countermeasure of hypercholesterolemia and has showed anti-inflammatory properties associated with the inhibition of Rho and Rho-associated kinases involved in a number of RI tissue injuries [65]. Pravastatin is known as an inhibitor of 3-hydroy-3 methyl-glutaryl-coenzyme A reductase (HMG-coA). However, although such property may explain its anticholesterolemiant action, the molecular mechanisms at the origin of its radioprotective effect remain unknown, inasmuch as some adverse side effects have been reported when statins are applied for a long period [66]. Some authors have suggested that statins may accelerate the DSB repair and can also increase the expression of some DNA repair proteins [67,68]. However, statins may induce ROS and some toxicities [69], so that they may be associated with antioxidative drugs. While its protective action in prostate cancer patients becomes more and more documented, no mechanistic model of the action of statins is still consensual [68].

In parallel, bisphosphonates in general, and zoledronate in particular, are currently used in treatment of osteoporosis and of cancer linked to hypercalcemia [70]. Bisphosphonates were shown to reduce skeletal events, notably pain, in the treatment against bone metastasis [71]. Bisphosphonates have the property to bind to exposed calcium areas and to cause apoptosis in osteoclasts [72]. Hence, like statins, while the molecular action of bisphosphonates is well-documented as a hypercalcemia countermeasure, no mechanistic model still explains its action in radioprotection.

A combined action of statins and aminobisphosphonates leads to the inhibition of both farnesylation and geranylgeranylation of progerin and prelamin A proteins, which are notably found cumulated in the nuclear membrane of progeroid cells [46]. Interestingly, these two drugs lead to increase the permeability of membranes, particularly those of mitochondria. With regard to statins, it is well known that cholesterol influences the organization of the membranes’ structure by inducing conformational ordering of the lipid chains: an anticholesterolemiant action may therefore impact the lipid layers’ permeability [73]. With regard to bisphosphonates, their impact on Ca^2+^ ions strongly suggests an important role in the regulation of the pores of the nuclear membranes [74]. Hence, to better document the radioprotective actions of each pro-episkevic drug alone and to compare them with the action of each antioxidative drug tested, it was useful to evaluate both applications of these drugs (i.e., alone or in association). Altogether, the literature and our data suggest that there is a body of evidence that a combination of statins and bisphosphonates would act on the protein trafficking between cytoplasm and nucleus. However, what is the link between such nucleoshuttling and radioprotection?

### 4.3. Toward a Unified Model for Chemical Radioprotection?

As evoked in the Introduction, the RIANS model is based on the occurrence of some important molecular steps [34]:

- DSB and ATM monomers induction: Immediately after irradiation, cytoplasmic ATM dimers dissociate in ATM monomers in a linearly dose-dependent manner; in parallel, in the nucleus, the RI DNA damage, and notably, the RI DSB, are induced in a dose-dependent manner as well [38]. During this step, the antioxidative approach leads to reduce the ROS in both cytoplasm and nucleus. Hence, the antioxidative approach would result in the decrease in the number of both RI-induced ATM monomers and DSB. This action should equally affect the DSB recognized by NHEJ and those recognized by any other pathways or even DSB that are not recognized at all. As a result, the number of early γH2AX foci decreases [31,32,33,40,41,48,49,50]. In the case of normal-RIANS quiescent cells, all the DSB are recognized by NHEJ: the number of γH2AX foci corresponds to the DSB physically induced by IR. Hence, the radioprotective effect of any antioxidative drug can be easily quantified on radioresistant controls. Conversely, in the case of delayed-RIANS quiescent cells, the difference between the number of DSB physically induced (about 40 per Gy) and the number of DSB recognized by NHEJ reflected by the γH2AX foci corresponds to the number of the DSB non-recognized by NHEJ or recognized by other pathways than NHEJ. At this step, the pro-episkevic drugs may not significantly impact the process.
- ATM monomers diffusion and DSB recognition: The RI ATM monomers diffuse in the nucleus. The pro-episkevic approach may result in accelerating and facilitating the diffusion of ATM monomers in the nucleus. Consequently, the number of pATM monomers that diffuse in the nucleus increases [31,32,33,40,41,48,49,50]. In the normal-RIANS quiescent cells, the number of ATM monomers available is sufficient to recognize all the DSB physically induced: no effect is observed. Conversely, in the delayed-RIANS quiescent cells, a larger number of ATM monomers contributes to recognize some additional DSB that are not recognized at all or that are recognized by other pathways than NHEJ. Hence, the number of γH2AX foci increases [31,32,33,40,41,48,49,50].
- DSB repair: In the case of the normal-RIANS quiescent cells, the pro-episkevic approach has no significant effect, while the antioxidative approach that leads to decrease the number of DSB does not reduce the proportion of unrepairable DSB. In the case of delayed-RIANS quiescent cells, the pro-episkevic approach contributes to increase the subset of DSB repaired by NHEJ, which decreases the RI lethal effect, while the antioxidative approach is not efficient enough to make the unrepaired DSB disappear.

The linear–quadratic (LQ) model is an empirical formula that links the cell survival to the IR dose. Since the 1970s, such a formula has been extensively used by radiobiologists and radiation oncologists to describe the individual response to IR [75]. The LQ model describes the clonogenic cell survival *S* as a function of dose *D* as follows:

(1) $$S=e^{-\left(\alpha D+\beta D^{2}\right)}$$

in which *α* and *β* are adjustable parameters to be determined [75].

**Figure 8:**
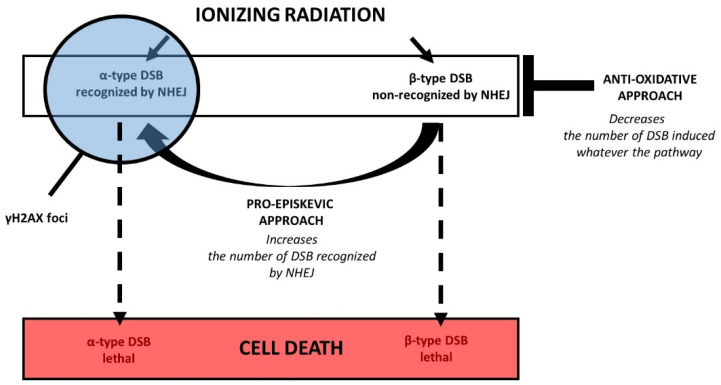
Schematic view of the molecular and cellular consequences of an exposure to IR inducing two types of DSB. The α-type DSB are recognized by the NHEJ DSB repair pathway, while the β-type DSB are not [38]. For each type of DSB, there are some subsets of unrepaired DSB [38]. Among them, some may be unrepairable and participate in the lethal effect. The γH2AX foci reveal the α-type DSB only. The antioxidative approach consists in reducing the number of DSB involved by IR, independently of the DSB repair pathways involved in their recognition. The pro-episkevic approach consists in increasing the number of DSB recognized by NHEJ by accelerating and facilitating the RIANS and therefore by stimulating the efficiency of NHEJ.

In 2016, the RIANS model provided a coherent interpretation of the two LQ parameters, α and β, by defining two types of RI and lethal DSB [38]: (1) The α-type DSB that are recognized by the ATM monomers in nucleus and therefore by NHEJ early after irradiation. Their number was demonstrated to be proportional to the dose [38]. (2) The β-type DSB that are not recognized by the ATM monomers in the nucleus (therefore not managed by NHEJ) because of a delay or an absence of the RIANS. Their number was demonstrated to be proportional to the square of the dose. It is noteworthy that the total number of DSB induced by IR (whatever their type, α or β) remains proportional to the dose [38]. A certain proportion of α- and β-type DSB may remain unrepairable and participate in the RI lethal effect. However, only the α-type DSB are detectable by γH2AX and pATM immunofluorescence. Hence, from the hypotheses developed above, the antioxidative approach results in the reduction in the number of DSB induced, whatever their type, α or β, while the pro-episkevic approach tends to transform the β-type DSB into α-type DSB (Figure 8). In other terms, the antioxidative approach may not change the mutation rate induced by IR, while the pro-episkevic approach may decrease it. Additional investigations are needed to verify such hypotheses.

## 5. Conclusions

In this study, we aimed to investigate and compare the molecular features of two different approaches of radioprotection. These investigations were performed, as a first step, with quiescent human fibroblasts and in standard conditions of a current RT session. Altogether, our findings suggest the following:

- The radiation protection strategy has long been based on an *antioxidative approach* consisting in reducing the oxidization process of the RI water radiolysis through an efficient reduction in ROS:
  ⚬ The number of DSB induced by IR decreases significantly;
  ⚬ Our findings confirm the efficiency of amifostine as one of the most efficient antioxidative drugs.
- The *pro-episkevic approach*, directly deriving from the RIANS model, aims at stimulating repair and contributes to increase the number of DSB managed by NHEJ—the most predominant DSB repair and signaling pathway in mammalians:
  ⚬ The combination of pravastatin and zoledronate (ZOPRA) shows a significant pro-episkevic property that appears to be more efficient than amifostine;
  ⚬ In the case of genetic syndromes associated with the loss of function of some genes essential for DSB repair, the effect of ZOPRA appears to be limited.

Additional investigations are needed to develop some new drugs that may elicit both antioxidative and pro-episkevic properties and to quantify the radiation protection action of both types of drugs applied together, whether on quiescent or proliferating cells. The question of the innocuity of long-term treatments should also be examined.

## Data Availability

All the data can be provided on reasonable request.

## References

[1] Foray N., Bourguignon M., Hamada N. (2016). Individual response to ionizing radiation. Mutat. Res. Rev..

[2] Weiss J.F., Landauer M.R. (2009). History and development of radiation-protective agents. Int. J. Radiat. Biol..

[3] Citrin D., Cotrim A.P., Hyodo F., Baum B.J., Krishna M.C., Mitchell J.B. (2010). Radioprotectors and mitigators of radiation-induced normal tissue injury. Oncologist.

[4] Mönig H., Messerschmidt O., Streffer C., Scherer E., Streffer C., Trott K.R. (1990). Chemical radioprotection in mammals and in man. Radiation Exposure and Occupational Risks.

[5] Lacassagne A. (1942). Chute de la sensiblité aux rayons X chez la souris nouveau-née en état d’asphyxie. C. R. L’académie Des. Sci..

[6] Akerfeldt S. (1959). Preparation and determination of sodium hydrogen S-(2-aminoethyl)-phosphorothioate. Acta Chem. Scand..

[7] Sweeney T.R. (1979). A Survey of Compounds from the Antiradiation Drug Development Program of the US Army Medical Research and Development Command.

[8] Walker R.I. (1988). Requirements of radioprotectors for military and emergency needs. Pharmacol. Ther..

[9] Restier-Verlet J., El-Nachef L., Ferlazzo M.L., Al-Choboq J., Granzotto A., Bouchet A., Foray N. (2021). Radiation on Earth or in Space: What Does It Change?. Int. J. Mol. Sci..

[10] Nair C.K., Parida D.K., Nomura T. (2001). Radioprotectors in radiotherapy. J. Radiat. Res..

[11] Weiss J.F. (1997). Pharmacologic approaches to protection against radiation-induced lethality and other damage. Environ. Health Perspect..

[12] Vachon A., Roman V., Lecomte C., Folcher G., Fatome M., Braquet P., Berleur F. (1987). A radioprotector: Cysteamine, inhibits oxygen transport in lipidic membranes. Int. J. Radiat. Biol. Relat. Stud. Phys. Chem. Med..

[13] Deschavanne P.J., Midander J., Debieu D., Malaise E.P., Revesz L. (1986). Radioprotective effect of cysteamine in glutathione synthetase-deficient cells. Int. J. Radiat. Biol. Relat. Stud. Phys. Chem. Med..

[14] Lespinasse F., Oiry J., Fatome M., Ardouin P., Imbach J., Malaise E.P., Guichard M. (1985). Radioprotection of EMT6 tumor by a new class of radioprotectors based on a pseudo-peptide cysteamine combination. Int. J. Radiat. Oncol. Biol. Phys..

[15] Bansal A., Simon M.C. (2018). Glutathione metabolism in cancer progression and treatment resistance. J. Cell Biol..

[16] Jadhav G.K., Bhanumathi P., Devi P.U., Seetharamaiah T., Vidyasagar M.S., Rao K.K., Hospet C.S., Solomon J.G. (1998). Possible role of glutathione in predicting radiotherapy response of cervix cancer. Int. J. Radiat. Oncol. Biol. Phys..

[17] Utley J.F., Phillips T.L., Kane L.J. (1976). Protection of normal tissues by WR2721 during fractionated irradiation. Int. J. Radiat. Oncol. Biol. Phys..

[18] Wadler S., Haynes H., Beitler J.J., Goldberg G., Holland J.F., Hochster H., Bruckner H., Mandeli J., Smith H., Runowicz C. (1993). Management of hypocalcemic effects of WR2721 administered on a daily times five schedule with cisplatin and radiation therapy. The New York Gynecologic Oncology Group. J. Clin. Oncol. Off. J. Am. Soc. Clin. Oncol..

[19] Treskes M., Boven E., van de Loosdrecht A.A., Wijffels J.F., Cloos J., Peters G.J., Pinedo H.M., van der Vijgh W.J. (1994). Effects of the modulating agent WR2721 on myelotoxicity and antitumour activity in carboplatin-treated mice. Eur. J. Cancer.

[20] Wynn R.B., Mehta V. (2005). Reduction of treatment breaks and radiation-induced esophagitis and pneumonitis using amifostine in unresectable non-small cell lung cancer patients receiving definitive concurrent chemotherapy and radiation therapy: A prospective community-based clinical trial. Semin. Oncol..

[21] King M., Joseph S., Albert A., Thomas T.V., Nittala M.R., Woods W.C., Vijayakumar S., Packianathan S. (2020). Use of Amifostine for Cytoprotection during Radiation Therapy: A Review. Oncology.

[22] Seed T.M., Inal C.E., Singh V.K. (2014). Radioprotection of hematopoietic progenitors by low dose amifostine prophylaxis. Int. J. Radiat. Biol..

[23] Singh V.K., Seed T.M. (2019). The efficacy and safety of amifostine for the acute radiation syndrome. Expert Opin. Drug Saf..

[24] Demirel C., Kilciksiz S., Ay O.I., Gurgul S., Ay M.E., Erdal N. (2009). Effect of N-acetylcysteine on radiation-induced genotoxicity and cytotoxicity in rat bone marrow. J. Radiat. Res..

[25] Samuni Y., Goldstein S., Dean O.M., Berk M. (2013). The chemistry and biological activities of N-acetylcysteine. Biochim. Biophys. Acta.

[26] Barlaz Us S., Vezir O., Yildirim M., Bayrak G., Yalin S., Balli E., Yalin A.E., Comelekoglu U. (2020). Protective effect of N-acetyl cysteine against radiotherapy-induced cardiac damage. Int. J. Radiat. Biol..

[27] Maasilta P., Holsti L.R., Blomqvist P., Kivisaari L., Mattson K. (1992). N-acetylcysteine in combination with radiotherapy in the treatment of non-small cell lung cancer: A feasibility study. Radiother. Oncol. J. Eur. Soc. Ther. Radiol. Oncol..

[28] Kryston T.B., Georgiev A.B., Pissis P., Georgakilas A.G. (2011). Role of oxidative stress and DNA damage in human carcinogenesis. Mutat. Res..

[29] Kurashige T., Shimamura M., Nagayama Y. (2017). N-Acetyl-L-cysteine protects thyroid cells against DNA damage induced by external and internal irradiation. Radiat. Environ. Biophys..

[30] Ferlazzo M. (2017). Impact of the Nucleoshuttling of the ATM Protein in Response to Ionizing Radiation: Notions of Pro- and Anti-Episkevic Features. PhD Thesis.

[31] Combemale P., Sonzogni L., Devic C., Bencokova Z., Ferlazzo M.L., Granzotto A., Burlet S.F., Pinson S., Amini-Adle M., Al-Choboq J. (2022). Individual Response to Radiation of Individuals with Neurofibromatosis Type I: Role of the ATM Protein and Influence of Statins and Bisphosphonates. Mol. Neurobiol..

[32] Ferlazzo M.L., Bach-Tobdji M.K.E., Djerad A., Sonzogni L., Burlet S.F., Devic C., Granzotto A., Bodgi L., Djeffal-Kerrar A., Foray N. (2017). Radiobiological characterization of tuberous sclerosis: A delay in the nucleo-shuttling of ATM may be responsible for radiosensitivity. Mol. Neurobiol..

[33] Bachelet J.T., Granzotto A., Ferlazzo M., Sonzogni L., Berthel E., Devic C., Foray N. (2021). First radiobiological characterization of the McCune-Albright syndrome: Influence of the ATM protein and effect of statins + bisphosphonates treatment. Int. J. Radiat. Biol..

[34] Berthel E., Foray N., Ferlazzo M.L. (2019). The Nucleoshuttling of the ATM Protein: A Unified Model to Describe the Individual Response to High- and Low-Dose of Radiation?. Cancers.

[35] Georgoulis A., Vorgias C.E., Chrousos G.P., Rogakou E.P. (2017). Genome Instability and gammaH2AX. Int. J. Mol. Sci..

[36] Berthel E., Ferlazzo M.C.D., Bourguignon M., Foray N. (2019). What does the History of Research on the Repair of DNA Double-Strand Breaks Tell Us?—A Comprehensive Review of Human Radiosensitivity. Int. J. Mol. Sci..

[37] Rothkamm K., Lobrich M. (2003). Evidence for a lack of DNA double-strand break repair in human cells exposed to very low x-ray doses. Proc. Natl. Acad. Sci. USA.

[38] Bodgi L., Foray N. (2016). The nucleo-shuttling of the ATM protein as a basis for a novel theory of radiation response: Resolution of the linear-quadratic model. Int. J. Radiat. Biol..

[39] Granzotto A., Benadjaoud M.A., Vogin G., Devic C., Ferlazzo M.L., Bodgi L., Pereira S., Sonzogni L., Forcheron F., Viau M. (2016). Influence of Nucleoshuttling of the ATM Protein in the Healthy Tissues Response to Radiation Therapy: Toward a Molecular Classification of Human Radiosensitivity. Int. J. Radiat. Oncol. Biol. Phys..

[40] Ferlazzo M.L., Sonzogni L., Granzotto A., Bodgi L., Lartin O., Devic C., Vogin G., Pereira S., Foray N. (2014). Mutations of the Huntington’s Disease Protein Impact on the ATM-Dependent Signaling and Repair Pathways of the Radiation-Induced DNA Double-Strand Breaks: Corrective Effect of Statins and Bisphosphonates. Mol. Neurobiol..

[41] Al-Choboq J., Ferlazzo M.L., Sonzogni L., Granzotto A., El-Nachef L., Maalouf M., Berthel E., Foray N. (2022). Usher Syndrome Belongs to the Genetic Diseases Associated with Radiosensitivity: Influence of the ATM Protein Kinase. Int. J. Mol. Sci..

[42] Le Reun E., Bodgi L., Granzotto A., Sonzogni L., Ferlazzo M.L., Al-Choboq J., El-Nachef L., Restier-Verlet J., Berthel E., Devic C. (2022). Quantitative correlations between radiosensitivity biomarkers show that the ATM protein kinase is strongly involved in the radiotoxicities observed after radiotherapy. Int. J. Mol. Sci..

[43] Joubert A., Zimmerman K.M., Bencokova Z., Gastaldo J., Rénier W., Chavaudra N., Favaudon V., Arlett C., Foray N. (2008). DNA double-strand break repair defects in syndromes associated with acute radiation response: At least two different assays to predict intrinsic radiosensitivity?. Int. J. Radiat. Biol..

[44] Foray N., Fertil B., Alsbeih M.G., Badie C., Chavaudra N., Iliakis G., Malaise E.P. (1996). Dose-rate effect on radiation-induced DNA double-strand breaks in the human fibroblast HF19 cell line. Int. J. Radiat. Biol..

[45] Foray N., Priestley A., Alsbeih G., Badie C., Capulas E.P., Arlett C.F., Malaise E.P. (1997). Hypersensitivity of ataxia telangiectasia fibroblasts to ionizing radiation is associated with a repair deficiency of DNA double-strand breaks. Int. J. Radiat. Biol..

[46] Varela I., Pereira S., Ugalde A.P., Navarro C.L., Suarez M.F., Cau P., Cadinanos J., Osorio F.G., Foray N., Cobo J. (2008). Combined treatment with statins and aminobisphosphonates extends longevity in a mouse model of human premature aging. Nat. Med..

[47] Foray N., Marot D., Gabriel A., Randrianarison V., Carr A.M., Perricaudet M., Ashworth A., Jeggo P. (2003). A subset of ATM- and ATR-dependent phosphorylation events requires the BRCA1 protein. EMBO J..

[48] Ferlazzo M., Berthel E., Granzotto A., Devic C., Sonzogni L., Bachelet J.T., Pereira S., Bourguignon M., Sarasin A., Mezzina M. (2019). Some mutations in the xeroderma pigmentosum D gene may lead to moderate but significant radiosensitivity associated with a delayed radiation-induced ATM nuclear localization. Int. J. Radiat. Biol..

[49] Bachelet J.T.A.G., Ferlazzo M., Sonzogni L., Berthel E.C.D., Foray N. (2020). First Radiobiological Characterization of Skin and Bone Cells from A Patient Suffering from the PI3KCA-Related Overgrowth Spectrum (PROS) Syndrome. Arch. Med. Clin. Case Rep..

[50] Moulay Lakhdar I., Ferlazzo M.L., Al Choboq J., Berthel E., Sonzogni L., Devic C., Granzotto A., Thariat J., Foray N. (2020). Fibroblasts from Retinoblastoma Patients Show Radiosensitivity Linked to Abnormal Localization of the ATM Protein. Curr. Eye Res..

[51] Badie C., Goodhardt M., Waugh A., Doyen N., Foray N., Calsou P., Singleton B., Gell D., Salles B., Jeggo P. (1997). A DNA double-strand break defective fibroblast cell line (180BR) derived from a radiosensitive patient represents a new mutant phenotype. Cancer Res..

[52] McKinnon P.J. (1987). Ataxia-telangiectasia: An inherited disorder of ionizing-radiation sensitivity in man. Progress in the elucidation of the underlying biochemical defect. Hum. Genet..

[53] Srinivas U.S., Tan B.W.Q., Vellayappan B.A., Jeyasekharan A.D. (2019). ROS and the DNA damage response in cancer. Redox Biol..

[54] Forman H.J., Zhang H., Rinna A. (2009). Glutathione: Overview of its protective roles, measurement, and biosynthesis. Mol. Asp. Med..

[55] Raghu G., Berk M., Campochiaro P.A., Jaeschke H., Marenzi G., Richeldi L., Wen F.Q., Nicoletti F., Calverley P.M.A. (2021). The Multifaceted Therapeutic Role of N-Acetylcysteine (NAC) in Disorders Characterized by Oxidative Stress. Curr. Neuropharmacol..

[56] Tenorio M., Graciliano N.G., Moura F.A., Oliveira A.C.M., Goulart M.O.F. (2021). N-Acetylcysteine (NAC): Impacts on Human Health. Antioxidants.

[57] Verhey L.J., Sedlacek R. (1983). Determination of the radioprotective effects of topical applications of MEA, WR-2721, and N-acetylcysteine on murine skin. Radiat. Res..

[58] Kataoka Y., Murley J.S., Baker K.L., Grdina D.J. (2007). Relationship between phosphorylated histone H2AX formation and cell survival in human microvascular endothelial cells (HMEC) as a function of ionizing radiation exposure in the presence or absence of thiol-containing drugs. Radiat. Res..

[59] Pedre B., Barayeu U., Ezerina D., Dick T.P. (2021). The mechanism of action of N-acetylcysteine (NAC): The emerging role of H2S and sulfane sulfur species. Pharmacol. Ther..

[60] Whillier S., Raftos J.E., Chapman B., Kuchel P.W. (2009). Role of N-acetylcysteine and cystine in glutathione synthesis in human erythrocytes. Redox Rep..

[61] Glover D., Riley L., Carmichael K., Spar B., Glick J., Kligerman M.M., Agus Z.S., Slatopolsky E., Attie M., Goldfarb S. (1983). Hypocalcemia and inhibition of parathyroid hormone secretion after administration of WR-2721 (a radioprotective and chemoprotective agent). N. Engl. J. Med..

[62] Akbulut S., Elbe H., Eris C., Dogan Z., Toprak G., Otan E., Erdemli E., Turkoz Y. (2014). Cytoprotective effects of amifostine, ascorbic acid and N-acetylcysteine against methotrexate-induced hepatotoxicity in rats. World J. Gastroenterol..

[63] Onalan G., Gulumser C., Mulayim B., Dagdeviren A., Zeyneloglu H. (2014). Effects of amifostine on endometriosis, comparison with N-acetyl cysteine, and leuprolide as a new treatment alternative: A randomized controlled trial. Arch. Gynecol. Obstet..

[64] Murray D., Prager A., Milas L. (1989). Radioprotection of cultured mammalian cells by the aminothiols WR-1065 and WR-255591: Correlation between protection against DNA double-strand breaks and cell killing after gamma radiation. Radiat. Res..

[65] Haydont V., Bourgier C., Pocard M., Lusinchi A., Aigueperse J., Mathe D., Bourhis J., Vozenin-Brotons M.C. (2007). Pravastatin Inhibits the Rho/CCN2/extracellular matrix cascade in human fibrosis explants and improves radiation-induced intestinal fibrosis in rats. Clin. Cancer Res. Off. J. Am. Assoc. Cancer Res..

[66] Balasubramanian R., Maideen N.M.P. (2021). HMG-CoA Reductase Inhibitors (Statins) and their Drug Interactions Involving CYP Enzymes, P-glycoprotein and OATP Transporters-An Overview. Curr. Drug Metab..

[67] Ziegler V., Henninger C., Simiantonakis I., Buchholzer M., Ahmadian M.R., Budach W., Fritz G. (2017). Rho inhibition by lovastatin affects apoptosis and DSB repair of primary human lung cells in vitro and lung tissue in vivo following fractionated irradiation. Cell Death Dis..

[68] Efimova E.V., Ricco N., Labay E., Mauceri H.J., Flor A.C., Ramamurthy A., Sutton H.G., Weichselbaum R.R., Kron S.J. (2018). HMG-CoA Reductase Inhibition Delays DNA Repair and Promotes Senescence After Tumor Irradiation. Mol. Cancer Ther..

[69] Sehitoglu I., Tumkaya L., Bedir R., Kalkan Y., Cure M.C., Yucel A.F., Zorba O.U., Yuce S., Cure E. (2015). Zoledronic acid aggravates kidney damage during ischemia reperfusion injury in rat. J. Environ. Pathol. Toxicol. Oncol..

[70] Mirrakhimov A.E. (2015). Hypercalcemia of Malignancy: An Update on Pathogenesis and Management. N. Am. J. Med. Sci..

[71] Wei Z., Pan B., Jia D., Yu Y. (2022). Long-term safety and efficacy of bisphosphonate therapy in advanced lung cancer with bone metastasis. Future Oncol..

[72] Srivichit B., Thonusin C., Chattipakorn N., Chattipakorn S.C. (2022). Impacts of bisphosphonates on the bone and its surrounding tissues: Mechanistic insights into medication-related osteonecrosis of the jaw. Arch. Toxicol..

[73] Zaborowska M., Broniatowski M., Wydro P., Matyszewska D., Bilewicz R. (2020). Structural modifications of lipid membranes exposed to statins—Langmuir monolayer and PM-IRRAS study. J. Mol. Lipids.

[74] Bootman M.D., Fearnley C., Smyrnias I., MacDonald F., Roderick H.L. (2009). An update on nuclear calcium signalling. J. Cell Sci..

[75] Bodgi L., Canet A., Pujo-Menjouet L., Lesne A., Victor J.M., Foray N. (2016). Mathematical models of radiation action on living cells: From the target theory to the modern approaches. A historical and critical review. J. Theor. Biol..

